# New monoclinic form of {*O*-Ethyl *N*-(4-nitro­phen­yl)thio­carbamato-κ*S*}(tri-4-tolyl­phosphane-κ*P*)gold(I): crystal structure and Hirshfeld surface analysis

**DOI:** 10.1107/S2056989017012865

**Published:** 2017-09-15

**Authors:** Fong Sheen Kuan, Mukesh M. Jotani, Edward R. T. Tiekink

**Affiliations:** a Department of Chemistry, National University of Singapore, 3 Science Drive 3, Singapore 117543; b Department of Physics, Bhavan’s Sheth R. A. College of Science, Ahmedabad, Gujarat 380001, India; c Research Centre for Crystalline Materials, School of Science and Technology, Sunway University, 47500 Bandar Sunway, Selangor Darul Ehsan, Malaysia

**Keywords:** crystal structure, gold, thiol­ate, polymorph, conformation, Hirshfeld surface analysis

## Abstract

The title phosphanegold(I) thiol­ate compound, [Au(C_9_H_9_N_2_O_3_S)(C_21_H_21_P)], is a second monoclinic polymorph (space group *P*2_1_/*c*) that complements a previously reported *Cc* polymorph [Broker & Tiekink (2008 ▸). *Acta Cryst*. E**64**, m1582]. An SP donor set defines an approximately linear geometry about the gold atom in both forms. The key distinguishing feature between the present structure and the previously reported polymorph rests with the relative disposition of the thiol­ate ligand. In the title compound, the orientation is such to place the oxygen atom in close contact with the gold atom [Au⋯O = 2.915 (2) Å], in contrast to the aryl ring in the original polymorph. In the crystal, linear supra­molecular chains along the *a*-axis direction mediated by C–H⋯π and nitro-O⋯π inter­actions are found. These pack with no directional inter­actions between them. The analysis of the Hirshfeld surfaces for both forms of [Au(C_9_H_9_N_3_O_3_S)(C_21_H_21_P)] indicates quite distinctive inter­action profiles relating to the differences in inter­molecular contacts found in their respective crystals.

## Abstract 2

A linear SP coordination geometry for the gold atom is found in the title structure, which also features a short intra­molecular Au⋯O inter­action, in contrast to a Au⋯π inter­action found in the first polymorph.

## Chemical context   

Phosphanegold(I) thiol­ates of the general formula *R*_3_PAu[SC(O*R*′)=N*R*′′], for *R*, *R*′ = alkyl, aryl and *R*′′ = aryl, have proven to exhibit exciting biological activities. For example, compounds of the type Ph_3_PAu[SC(O*R*)=NPh], *R* = Me, Et and *i*-Pr, induce G_2_/M cell cycle arrest in HT-29 cancer cells and exhibit tolerable toxicity based on experiments on zebrafish (Yeo, Ooi *et al.*, 2013 ▸; Ooi *et al.*, 2017 ▸). Further, *in vitro* mechanistic investigations point to these compounds inducing both intrinsic and extrinsic pathways of cell death leading to apoptosis. In complementary studies on compounds with *R*′′ = 4-tolyl, quite promising *in vitro* potency against Gram-positive bacteria has been revealed (Yeo, Sim *et al.*, 2013 ▸). However, such biological potential does not extend to activity against certain *Acanthamoeba castellanii* (Siddiqui *et al.*, 2017 ▸). The observed biological activity for this class of compound has necessitated synthesis and re-synthesis during the course of which various polymorphs (*e.g*. Yeo *et al.*, 2016*a* ▸) and solvates (*e.g*. Yeo *et al.*, 2016*b* ▸) have been revealed. Of particular inter­est has been the recent appearance of conformational polymorphs for these compounds.

Referring the conformation shown in the Scheme, most structures having the formula *R*_3_PAu[SC(O*R*′)=N*R*′′] display an intra­molecular Au⋯O inter­action. In an exercise in crystal engineering, it was argued that by moderating the electronic properties of the phosphane-bound and thiol­ate-*N*-bound groups, it was possible to direct a change in conformation so that an intra­molecular Au⋯π(ar­yl) inter­action formed instead of the Au⋯O contact (Kuan *et al.*, 2008 ▸). Such Au⋯π(ar­yl) inter­actions are well established in the supra­molecular chemistry of mol­ecular gold compounds (Tiekink & Zukerman-Schpector, 2009 ▸; Caracelli *et al.*, 2013 ▸) and have important implications in mechanisms associated with catalytic gold (Lin & Hammond, 2012 ▸). As mentioned above, current inter­est in the biological activity of this class of compounds has prompted renewed synthesis and scale-up. Recently, a conformational polymorph was discovered during a check for sample purity, *via* powder X-ray diffraction, for a compound, Ph_3_PAu[SC(OEt)=NPh], that was originally reported in a form with an intra­molecular Au⋯O inter­action (Hall & Tiekink, 1993 ▸). The new polymorph featured an intra­molecular Au⋯π(ar­yl) inter­action instead, an observation ascribed to thermodynamic considerations (Yeo, Tan, Otero-de-la-Roza *et al.*, 2016 ▸). Herein, as a continuation of structural studies of these compounds, a new polymorph for (4-tol)_3_PAu[SC(OEt)=NC_6_H_4_NO_2_-4] is reported which was reported originally in space group *Cc* with a Au⋯π(ar­yl) inter­action (Broker & Tiekink, 2008 ▸), but now with a Au⋯O inter­action. Herein, the crystal and mol­ecular structures of a *P*2_1_/*c* polymorph of (4-tol)_3_PAu[SC(OEt)=NC_6_H_4_NO_2_-4], (I), are described complemented by an analysis of the Hirshfeld surfaces calculated for (I) and for the original *Cc* form, (II).
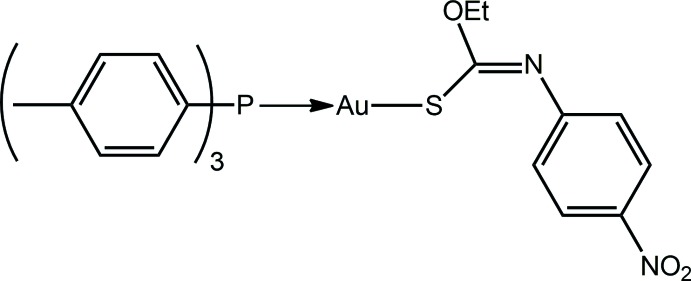


## Structural commentary   

**Figure 1:**
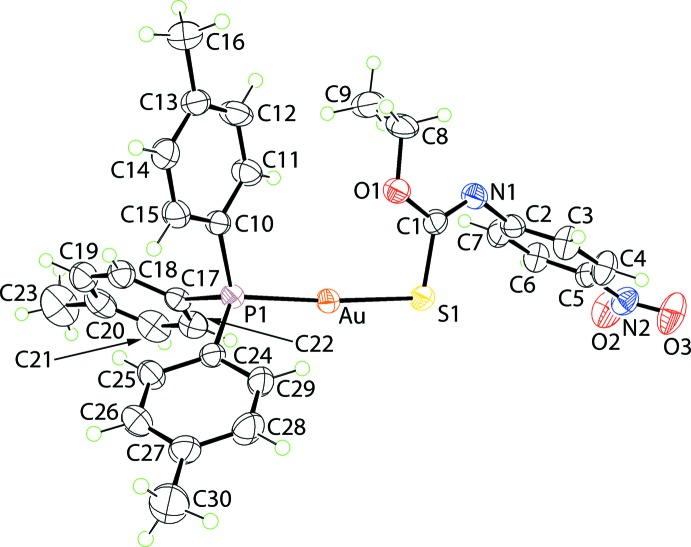
The mol­ecular structure of (I) showing the atom-labelling scheme and displacement ellipsoids at the 50% probability level.

The mol­ecular structure of (I) is shown in Fig. 1 ▸ and selected inter­atomic parameters are collected in Table 1 ▸. The gold(I) atom is coordinated by thiol­ate-S and phosphane-P atoms in a near linear geometry. The P1—Au—S angle of 175.80 (3)° deviates from the ideal 180°, an observation which might be ascribed to the formation of an intra­molecular Au⋯O inter­action of 2.915 (2) Å, which arises as the thiol­ate ligand is orientated to place the oxygen atom in close proximity to the gold atom. As is usual for these compounds, the Au—S bond is longer than the Au—P bond. The C1=N1 bond length of 1.259 (4) Å is consistent with significant double character in this bond and, by implication, the presence of a thiol­ate-*S* atom. These bond-length conclusions are vindicated by a comparison of the bond lengths found in the uncoordinated mol­ecule, *i.e*. EtOC(=S)N(H)C_6_H_4_NO_2_-4 (Benson *et al.*, 2006 ▸). Here, the C1=S1 and C1—N1 bond lengths are 1.672 (2) and 1.354 (3) Å, respectively, *i.e*. clearly shorter and longer than the related bond lengths in (I). The equivalent geometric parameters to those listed in Table 1 ▸ for the *Cc* polymorph (Broker & Tiekink, 2008 ▸) are equal within experimental error with one possible exception, being the P—Au—S angle, which at 174.54 (10)° appears to be narrower by about 1° than the equivalent angle in (I), Table 1 ▸.

The central S1, O1, N1 and C1 atoms of the thiol­ate ligand are strictly (r.m.s. deviation of the fitted atoms = 0.0008 Å) planar. The plane through the nitro­benzene ligand is orthogonal to the former plane, forming a dihedral angle of 89.67 (12)°. Finally, the nitro group is essentially co-planar with the ring to which it is connected, forming a dihedral angle of 4.7 (4)°.

**Figure 2:**
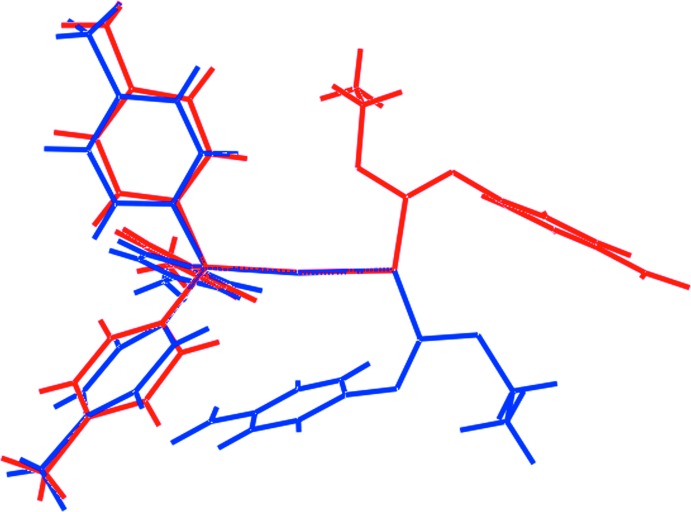
Overlay diagram of the mol­ecular structures found in (I) (*P*2_1_/*c*, red image) and (II) (*Cc*, blue) forms of (4-tol)_3_PAu[SC(OEt)=NC_6_H_4_NO_2_-4]. The mol­ecules have been overlapped so that the P—Au—S fragments are coincident.

The differences in conformation for (I) and (II) are starkly highlighted in the overlay diagram shown in Fig. 2 ▸. Some physical properties for the two forms, calculated in *Crystal Explorer* (Wolff *et al.*, 2012 ▸) and *PLATON* (Spek, 2009 ▸), are included in Table 2 ▸. These data indicate significant differences between the mol­ecules comprising polymorphs (I) and (II), most notably indicating the mol­ecule in (II) to be more compact, spherical and to have a greater density, all parameters consistent with this being the thermodynamically more stable form.

## Supra­molecular features   

**Figure 3:**
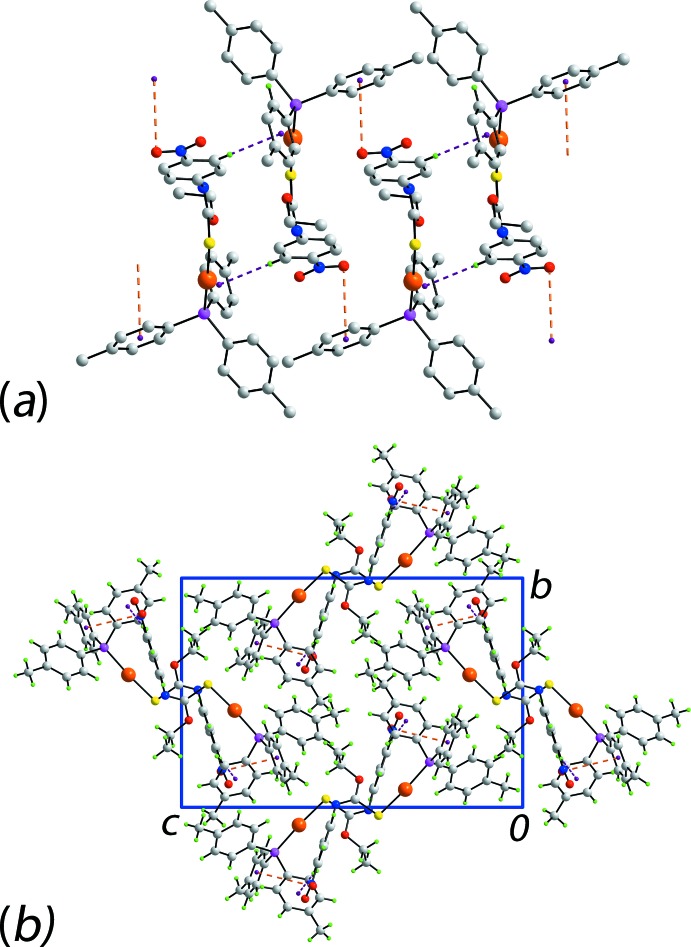
Mol­ecular packing in (I): (*a*) a view of the linear supra­molecular chain sustained by nitro­benzene-C—H⋯π(tol­yl) inter­actions as well as nitro-O⋯π(tol­yl) contacts shown as purple and orange dashed lines, respectively (non-participating H atoms have been removed) and (*b*) a view of the unit-cell contents shown in projection down the *a* axis.

The geometric parameters defining the identified inter­molecular inter­actions are listed in Table 3 ▸. The key feature of the mol­ecular packing is the formation of linear supra­molecular chains along the *a*-axis direction, Fig. 3 ▸ *a*. These are sustained by a combination of nitro­benzene-C—H⋯π(tol­yl) inter­actions as well as nitro-O⋯π(tol­yl) contacts, Fig. 3 ▸ *b*. For the latter, the nitro group lies over the ring, with the two residues being almost parallel, forming a dihedral angle = 7.4 (2)°. While comparatively rare, the latter inter­actions have been discussed in the crystallographic literature (Huang *et al.*, 2008 ▸).

## Analysis of the Hirshfeld surfaces   

The Hirshfeld surface calculations on polymorphic (I) and (II) were performed in accord with recent related work (Jotani *et al.*, 2017 ▸). In short, the two monoclinic polymorphs reveal quite distinctive features in their Hirshfeld surfaces.

**Figure 4:**
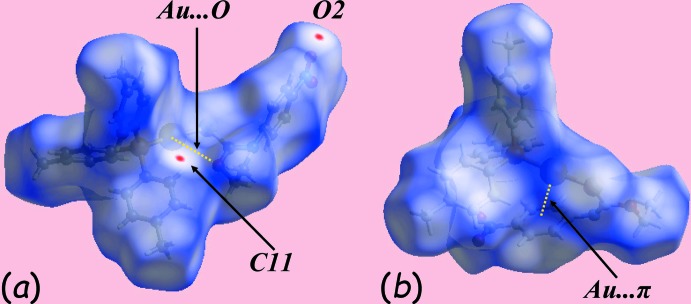
Views of the Hirshfeld surface mapped over *d*_norm_ for (*a*) (I) in the range −0.003 to +1.441 au and (*b*) (II) in the range 0.007 to 1.513 au.

**Figure 5:**
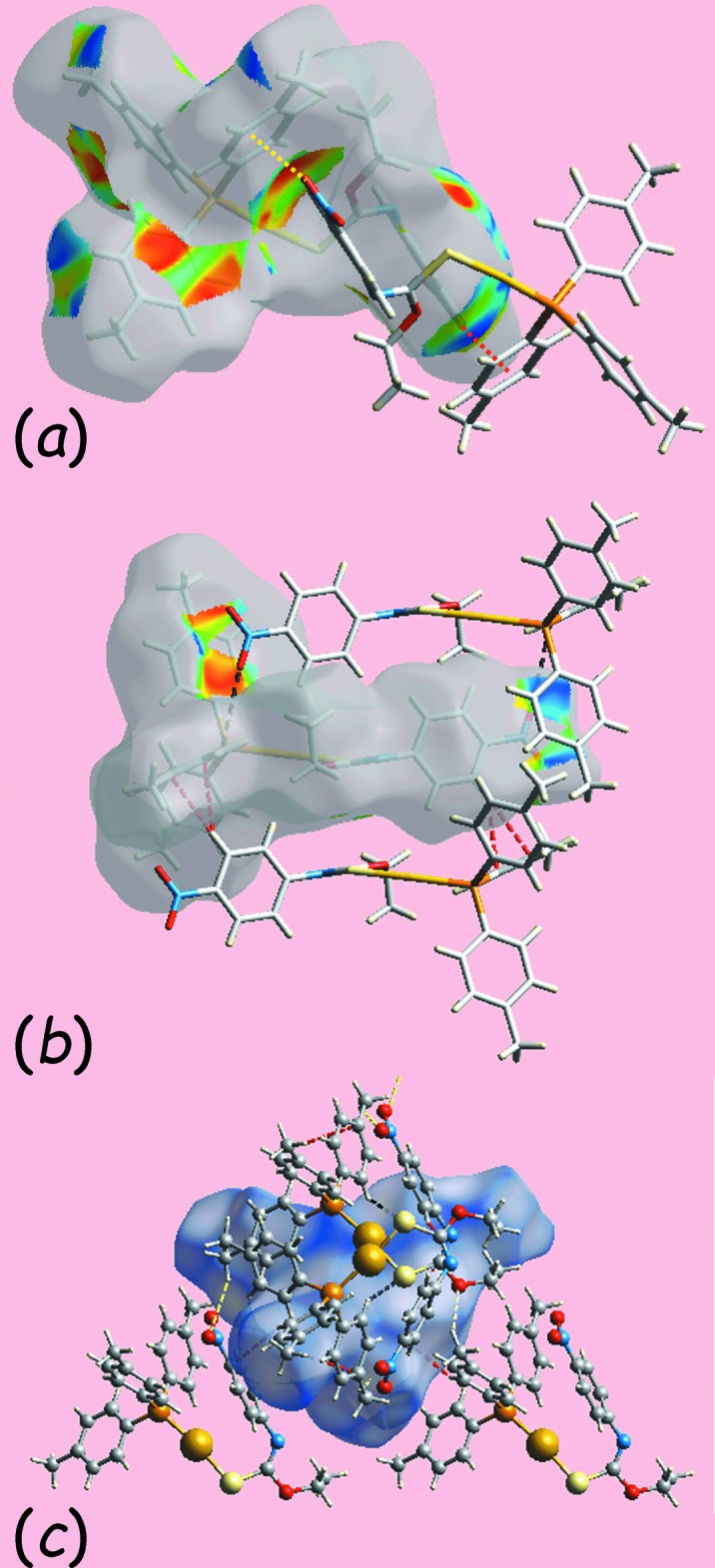
Views of the Hirshfeld surfaces about a reference mol­ecule mapped over (*a*) the shape-index property for (I) showing C—H⋯π and its reciprocal, *i.e*. π⋯H—C, contacts as red and yellow dotted line, respectively, (*b*) the shape-index property for (I) showing short inter-atomic C⋯O/O⋯C and C⋯H/H⋯C contacts as black and red dashed lines, respectively, and (*c*) over *d*_norm_ for (II) showing short inter-atomic C⋯H/H⋯C, S⋯H/H⋯S and O⋯H/H⋯O contacts as red, black and yellow dashed lines, respectively.

It is clearly evident from the Hirshfeld surfaces mapped over *d*_norm_ for forms (I) and (II), Fig. 4 ▸, that the former conformation favours an intra­molecular Au⋯O contact while the latter, an intra­molecular Au⋯π(ar­yl) inter­action. In addition, the tiny red spots appearing near the nitro-O2 and tolyl-C11 atoms in Fig. 4 ▸ *a* indicate the significance of short inter-atomic C⋯O/O⋯C contacts, Table 4 ▸, in the packing of (I). The immediate environments about a reference mol­ecule within the shape-index mapped surface for (I), Fig. 5 ▸ *a*, *b*, and the *d*_norm_-mapped surface for (II), Fig. 5 ▸ *c*, are consistent with (I) forming C—H⋯π and N—O⋯π inter­actions together with few short inter-atomic contacts in its packing, whereas the packing of (II) involves only a few short inter-atomic contacts, Table 4 ▸. The donor and acceptor of the C—H⋯π(ar­yl) contact in (I) appear as blue and bright-red regions around the participating atoms and highlighted with red and yellow dotted lines in Fig. 5 ▸ *a*. The inter­molecular nitro-O⋯π inter­action involving both nitro­benzene-O2 and O3 atoms with the same symmetrically located tolyl ring (C17–C22) are viewed as two adjoining blue and bright-orange regions in Fig. 5 ▸ *b*. The short inter-atomic S⋯H/H⋯S, C⋯H/H⋯C and O⋯H/H⋯O contacts influential in the structure of (II) are highlighted with black, red and yellow dashed lines, respectively, in Fig.5*c*.

**Figure 6:**
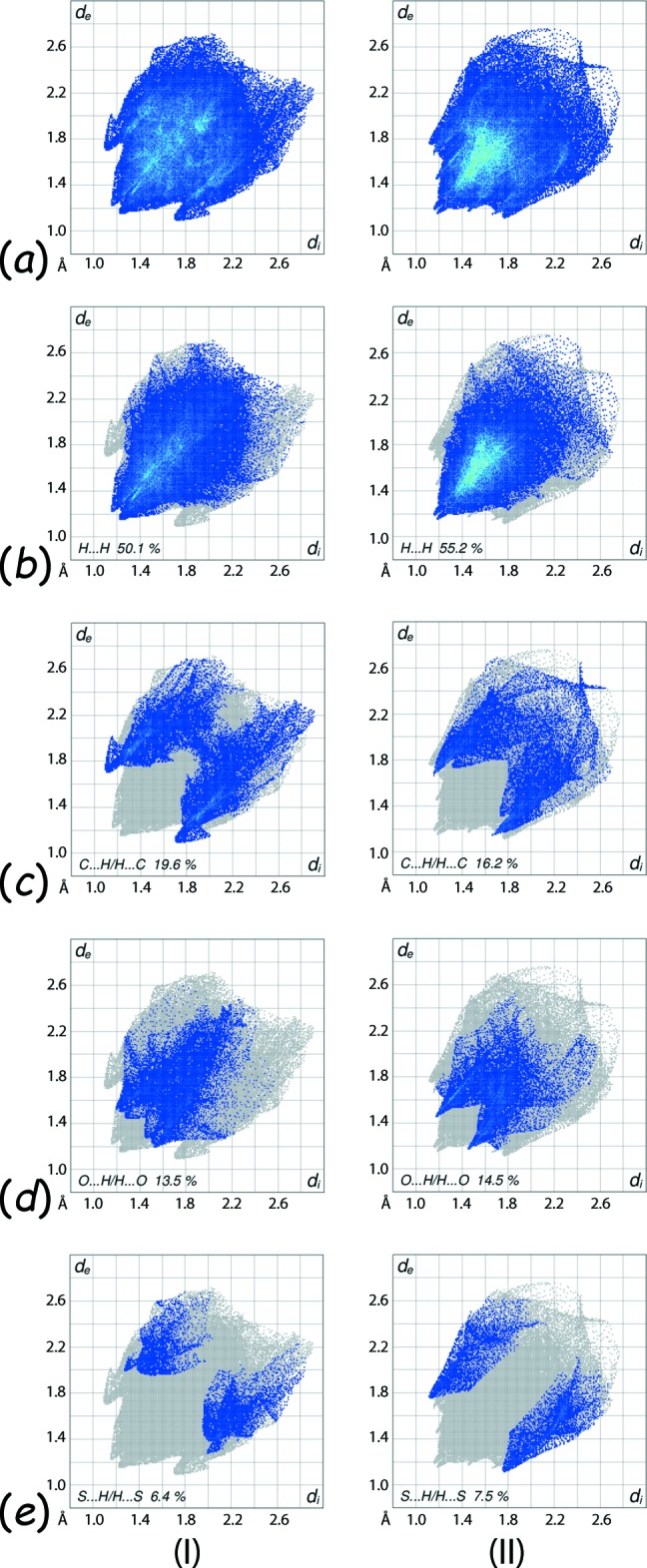
(*a*) The full two-dimensional fingerprint plots for (I) and (II), and those delineated into (*b*) H⋯H, (*c*) C⋯H/H⋯C, (*d*) O⋯H/H⋯O and (*e*) S⋯H/H⋯S contacts.

From the overall two-dimensional fingerprint plots for (I) and (II), Fig. 6 ▸ *a*, it is apparent that the different orientations of the thiol­ate ligands significantly impact upon the observed features in the plots. This is also visible from the fingerprints delineated into H⋯H, C⋯H/H⋯C, O⋯H/H⋯O and S⋯H/H⋯S contacts (McKinnon *et al.*, 2007 ▸) in Fig. 6 ▸ *b*–*e*, and in the relative percentage contributions from the different contacts to the Hirshfeld surfaces, as summarized in Table 5 ▸. Although H⋯H contacts make dominant contributions of 50.1 and 55.2% to the Hirshfeld surfaces of (I) and (II), respectively, the plot area and the distribution of characteristic points within the plots indicate different propensities to form such inter-atomic contacts, Fig. 6 ▸ *b*. The pair of small, closely situated peaks at *d*_e_ + *d*_i_ < 2.40 Å, *i.e.* the sum of two times the van der Waals radius of hydrogen, are observed for both the polymorphs and reflect short inter-atomic H⋯H contacts, Table 4 ▸.

The distinctive features of fingerprint plot delineated into C⋯H/H⋯C contacts, Fig. 6 ▸ *c*, wherein the half-arrows in (I) contrast the forceps in (II) with their tips at *d*_e_ + *d*_i_ ∼ 2.8 Å and 2.9 Å, respectively, arise as the result of distinctive inter­molecular inter­actions in the two forms: the former has a C—H⋯π contact while the latter has short inter-atomic C⋯H/H⋯C contacts, Fig. 5 ▸ *c* and Table 4 ▸. Thus, the short C⋯H/H⋯C contacts involving the nitro­benzene-H4 atom inter­acting with the tolyl-C12 and C13 atoms for (I), Table 4 ▸, have analogous contacts in form (II), Fig. 5 ▸ *b* and Table 4 ▸. Although, O⋯H/H⋯O and S⋯H/H⋯S contacts make almost similar percentage contributions to the Hirshfeld surfaces for both the forms, Table 4 ▸, the distinct features in their delineated fingerprint plots, Fig. 6 ▸ *c* and *d*, reflects the different types of inter-atomic contacts they form. In the respective plots for the form (I), the distribution of characteristic points are far away from the van der Waals separations indicating the absence of such short inter-atomic contacts in the packing. By contrast, the forceps-like tips at *d*_e_ + *d*_i_ ∼2.7 Å in the O⋯H/H⋯O delineated and the knife-edge tips at *d*_e_ + *d*_i_ ∼2.9 Å in the S⋯H/H⋯S delineated fingerprint plots for (II) are the result of short inter-atomic O⋯H/H⋯O and S⋯H/H⋯S contacts, Table 4 ▸. The other inter-atomic contacts summarized in Table 4 ▸ have small percentage contributions to the Hirshfeld surfaces of (I) and (II) and are considered to have negligible influence in the crystals.

## Database survey   

A measure of the significance of Au⋯π(ar­yl) inter­actions can be seen in the polymorphic structures of ClAuP(Ph_2_)CH_2_(Ph_2_)PAuCl. In the original form, intra­molecular Au⋯Au inter­actions [3.34 Å] were observed (Schmidbaur *et al.*, 1977 ▸) but, in the more recently determined second form, intra­molecular Au⋯π(ar­yl) inter­actions (3.58 Å) were formed instead (Healy, 2003 ▸). The real significance of this is that the energy of stabilization to a structure provided by Au⋯Au inter­actions is comparable to that provided by conventional hydrogen bonding (Schmidbaur, 2001 ▸). This observation lead to systematic investigations into the cooperation/competition between hydrogen-bonding and Au⋯Au inter­actions (Schneider *et al.*, 1996 ▸; Schmidbaur *et al.*, 2012 ▸) with the former often winning out owing to steric pressures associated with bringing gold centres into close proximity (Tiekink, 2014 ▸). The structures found for ClAuP(Ph_2_)CH_2_(Ph_2_)PAuCl imply that Au⋯π(ar­yl) inter­actions provide comparable energies of stabilization to their crystal structures. Indeed, computational chemistry on the polymorphic system Ph_3_PAu[SC(OEt)=NPh] suggested the form with the intra­molecular Au⋯π(ar­yl) contact was more than 5 kcal  mol^−1^ stable than the form with the intra­molecular Au⋯O contact (Yeo *et al.*, 2015 ▸). Related studies on a binuclear compound of the general formula [Et_3_PAuS(OMe)=N]_2_(1,4-C_6_H_4_) indicated that each Au⋯π(ar­yl) inter­action in the centrosymmetric mol­ecule was more stable by more than 12 kcal mol^−1^ than each putative Au⋯O contact (Yeo *et al.*, 2015 ▸). This near equivalence in energies of different inter­molecular contacts in metal-containing species is the focus of a recent review (Tiekink, 2017 ▸).

## Synthesis and crystallization   

The title compound (I) was prepared following established literature procedures (Ho *et al.*, 2006 ▸). Yellow crystals were obtained by the slow evaporation of a CH_2_Cl_2_/Et_2_O/hexane (1:1:2) solution of (I). Crystals with the same unit-cell characteristics were also isolated from benzene and ethyl­acetate solutions of (I). ^1^H NMR (δ): thiol­ate: 7.92 (*d*, Ha, *J* = 8.8 Hz), 6.89 (*d*, Hb, *J* = 8.8 Hz), 4.34 (*q*, OCH_2_, *J* = 7.2 Hz), 1.35 (*t*, CH_3_, *J* = 7.2 Hz). Phosphane: 7.32–7.22 (*m*, aryl-H), 2.40 (*s*, Me). ^13^C NMR (δ): Thiol­ate: 165.7 (*s*, C_q_), 157.5 (*s*, C_1_), 142.6 (*s*, C_4_), 124.8 (*s*, C_3_), 122.5 (*s*, C_2_), 64.5 (*s*, OCH_2_), 14.5 (*s*, CH_3_). Phosphane: 142.2 (*s*, C_δ_), 133.9 (*d*, C_β_, *J* = 14.2 Hz), 129.8 (*d*, C_γ_, *J* = 12.0 Hz), 126.4 (*d*, C_α_, *J* = 58.2 Hz), 21.4 (*s*, Me).

## Refinement   

Crystal data, data collection and structure refinement details are summarized in Table 6 ▸. The carbon-bound H atoms were placed in calculated positions (C—H = 0.94–0.98 Å) and were included in the refinement in the riding model approximation, with *U*_iso_(H) set to 1.2–1.5*U*_eq_(C). The maximum and minimum residual electron density peaks of 1.16 and 0.78 e Å^−3^, respectively, were located 0.81 and 1.28 Å from the Au atom. Owing to inter­ference from the beam-stop, the (011) reflection was omitted from the final cycles of refinement.

## Supplementary Material

Crystal structure: contains datablock(s) I, global. DOI: 10.1107/S2056989017012865/hb7703sup1.cif

Structure factors: contains datablock(s) I. DOI: 10.1107/S2056989017012865/hb7703Isup2.hkl

CCDC reference: 1573275

Additional supporting information:  crystallographic information; 3D view; checkCIF report

## Figures and Tables

**Table 1:** Selected geometric parameters (Å, °)

Au—P1	2.2611 (8)	S1—C1	1.756 (3)
Au—S1	2.3105 (8)	N1—C1	1.259 (4)
			
P1—Au—S1	175.80 (3)	C1—S1—Au	100.18 (11)

**Table 2:** A comparison of some physical properties between the mol­ecules in the polymorphs of (4-tol)_3_PAu[SC(OEt)=NC_6_H_4_NO_2_-4]

Mol­ecule	Volume, *V* (Å^3^)	Area, *A* (Å^2^)	*A*:*V*	Globularity, *G*	Asphericity, *Ω*	*D*_*x*_’
*P*2_1_/*c* form, (I)	714.31	603.78	0.845	0.640	0.117	1.663
*Cc* form, (II)	698.76	531.90	0.761	0.716	0.036	1.704

**Table 3:** Hydrogen-bond geometry (Å, °) *Cg*1 and *Cg*2 are the centroids of the C10–C15 and C17–C22 rings, respectively.

*D*—H⋯*A*	*D*—H	H⋯*A*	*D*⋯*A*	*D*—H⋯*A*
C4—H4⋯*Cg*1^i^	0.94	2.64	3.528 (4)	157
N2—O2⋯*Cg*2^ii^	1.22 (1)	3.55 (1)	83.1 (2)	4 (1)
N2—O3⋯*Cg*2^ii^	1.21 (1)	3.83 (1)	70.2 (2)	4 (1)

Symmetry codes: (i) 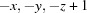; (ii) 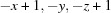.

**Table 4:** Summary of short inter-atomic contacts (Å) in (I) and (II)

Contact	Distance	Symmetry operation
(I)		
O2⋯C11	3.175 (5)	1 − *x*, −*y*, 1 − *z*
H4⋯C12	2.89	−*x*, −*y*, 1 − *z*
H4⋯C13	2.81	−*x*, −*y*, 1 − *z*
H9*B*⋯H16*B*	2.38	−*x*, 1 − *y*, 1 − *z*
(II)		
S1⋯H3A	2.97	*x*, 1 − *y*, − 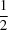 + *z*
S1⋯H25A	2.88	*x*, 1 − *y*, 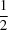 + *z*
C4⋯H18A	2.89	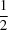 + *x*, 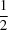 − *y*, 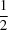 + *z*
C7⋯H2A	2.82	*x*, 1 − *y*, − 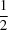 + *z*
O2⋯H30*C*	2.63	*x*, *y*, 1 + *z*
H12A⋯H30*B*	2.35	− 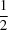 + *x*, 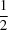 + *y*, *z*

**Table 5:** Percentage contributions of inter-atomic contacts to the Hirshfeld surfaces for (I)

		Percentage contribution
Contact	(I)	(II)
H⋯H	50.1	55.2
C⋯H/H⋯C	19.6	16.2
O⋯H/H⋯O	13.5	14.5
S⋯H/H⋯S	6.4	7.5
Au⋯H/H⋯Au	2.9	1.9
C⋯O/O⋯C	2.5	1.3
C⋯C	2.1	1.3
N⋯H/H⋯N	1.8	2.1
C⋯N/N⋯C	0.8	0.0
N⋯O /O⋯N	0.2	0.0
S⋯N/N⋯S	0.1	0.0

**Table 6:** Experimental details

Crystal data	
Chemical formula	[Au(C_9_H_9_N_2_O_3_S)(C_21_H_21_P)]
*M*_r_	726.55
Crystal system, space group	Monoclinic, *P*2_1_/*c*
Temperature (K)	223
*a*, *b*, *c* (Å)	9.8815 (6), 14.0448 (9), 21.2332 (13)
β (°)	99.924 (2)
*V* (Å^3^)	2902.7 (3)
*Z*	4
Radiation type	Mo *K*α
μ (mm^−1^)	5.23
Crystal size (mm)	0.27 × 0.12 × 0.11

Data collection	
Diffractometer	Bruker AXS *SMART* CCD
Absorption correction	Multi-scan (*SADABS*; Bruker, 2000 ▸)
*T*_min_, *T*_max_	0.421, 1
No. of measured, independent and observed [*I* > 2σ(*I*)] reflections	23909, 8412, 6748
*R*_int_	0.040
(sin θ/λ)_max_ (Å^−1^)	0.703

Refinement	
*R*[*F* ^2^ > 2σ(*F* ^2^)], *wR*(*F* ^2^), *S*	0.031, 0.078, 0.95
No. of reflections	8412
No. of parameters	347
H-atom treatment	H-atom parameters constrained
Δρ_max_, Δρ_min_ (e Å^−3^)	1.16, −0.78

Computer programs: *SMART* and *SAINT* (Bruker, 2000 ▸), *SHELXS97* (Sheldrick, 2008 ▸), *SHELXL2014/7* (Sheldrick, 2015 ▸), *ORTEP-3 for Windows* (Farrugia, 2012 ▸), *DIAMOND* (Brandenburg, 2006 ▸), *QMol* (Gans & Shalloway, 2001 ▸) and *publCIF* (Westrip, 2010 ▸).

## supplementary crystallographic information

### Crystal data

**Table:**

[Au(C_9_H_9_N_2_O_3_S)(C_21_H_21_P)]	*F*(000) = 1432
*M**_r_* = 726.55	*D*_x_ = 1.663 Mg m^−^^3^
Monoclinic, *P*2_1_/*c*	Mo *K*α radiation, λ = 0.71069 Å
*a* = 9.8815 (6) Å	Cell parameters from 7098 reflections
*b* = 14.0448 (9) Å	θ = 2.4–28.2°
*c* = 21.2332 (13) Å	µ = 5.23 mm^−^^1^
β = 99.924 (2)°	*T* = 223 K
*V* = 2902.7 (3) Å^3^	Block, yellow
*Z* = 4	0.27 × 0.12 × 0.11 mm

### Data collection

**Table:**

Bruker AXS SMART CCD diffractometer	8412 independent reflections
Radiation source: fine-focus sealed tube	6748 reflections with *I* > 2σ(*I*)
Graphite monochromator	*R*_int_ = 0.040
ω scans	θ_max_ = 30.0°, θ_min_ = 2.0°
Absorption correction: multi-scan (SADABS; Bruker, 2000)	*h* = −10→13
*T*_min_ = 0.421, *T*_max_ = 1	*k* = −19→19
23909 measured reflections	*l* = −29→19

### Refinement

**Table:**

Refinement on *F*^2^	0 restraints
Least-squares matrix: full	Hydrogen site location: inferred from neighbouring sites
*R*[*F*^2^ > 2σ(*F*^2^)] = 0.031	H-atom parameters constrained
*wR*(*F*^2^) = 0.078	*w* = 1/[σ^2^(*F*_o_^2^) + (0.0423*P*)^2^] where *P* = (*F*_o_^2^ + 2*F*_c_^2^)/3
*S* = 0.95	(Δ/σ)_max_ = 0.002
8412 reflections	Δρ_max_ = 1.16 e Å^−^^3^
347 parameters	Δρ_min_ = −0.78 e Å^−^^3^

### Special details

**Table:**

Geometry. All esds (except the esd in the dihedral angle between two l.s. planes) are estimated using the full covariance matrix. The cell esds are taken into account individually in the estimation of esds in distances, angles and torsion angles; correlations between esds in cell parameters are only used when they are defined by crystal symmetry. An approximate (isotropic) treatment of cell esds is used for estimating esds involving l.s. planes.

### Fractional atomic coordinates and isotropic or equivalent isotropic displacement parameters (Å^2^)

**Table:**

	*x*	*y*	*z*	*U*_iso_*/*U*_eq_	
Au	0.12283 (2)	0.07759 (2)	0.34429 (2)	0.03271 (5)	
S1	0.18283 (10)	−0.03609 (6)	0.42279 (4)	0.03903 (18)	
P1	0.06779 (8)	0.19705 (6)	0.27309 (4)	0.03035 (16)	
O1	0.1276 (2)	0.12051 (16)	0.47896 (10)	0.0395 (5)	
O2	0.6243 (3)	−0.3434 (2)	0.61518 (16)	0.0661 (8)	
O3	0.4384 (3)	−0.3994 (2)	0.63774 (18)	0.0711 (9)	
N1	0.2402 (3)	0.0118 (2)	0.54778 (13)	0.0419 (7)	
N2	0.5036 (3)	−0.3355 (2)	0.61897 (15)	0.0451 (7)	
C1	0.1877 (3)	0.0344 (2)	0.49148 (15)	0.0343 (6)	
C2	0.3048 (4)	−0.0766 (2)	0.56273 (15)	0.0368 (7)	
C3	0.2308 (4)	−0.1524 (3)	0.58156 (18)	0.0461 (8)	
H3	0.1361	−0.1459	0.5815	0.055*	
C4	0.2960 (4)	−0.2371 (3)	0.60035 (19)	0.0473 (9)	
H4	0.2462	−0.2887	0.6129	0.057*	
C5	0.4348 (3)	−0.2452 (2)	0.60053 (15)	0.0356 (7)	
C6	0.5105 (3)	−0.1700 (3)	0.58361 (17)	0.0422 (8)	
H6	0.6056	−0.1763	0.5848	0.051*	
C7	0.4448 (4)	−0.0855 (2)	0.56491 (19)	0.0449 (8)	
H7	0.4956	−0.0335	0.5536	0.054*	
C8	0.1369 (4)	0.1857 (3)	0.53290 (17)	0.0505 (10)	
H8A	0.1311	0.1498	0.5719	0.061*	
H8B	0.0599	0.2307	0.5254	0.061*	
C9	0.2709 (5)	0.2398 (3)	0.5413 (2)	0.0596 (11)	
H9A	0.3469	0.1951	0.5477	0.089*	
H9B	0.2776	0.2812	0.5783	0.089*	
H9C	0.2744	0.2777	0.5035	0.089*	
C10	−0.0001 (3)	0.2979 (2)	0.31120 (14)	0.0312 (6)	
C11	0.0534 (4)	0.3172 (3)	0.37427 (16)	0.0419 (8)	
H11	0.1214	0.2772	0.3967	0.050*	
C12	0.0079 (4)	0.3948 (3)	0.40474 (17)	0.0483 (9)	
H12	0.0464	0.4074	0.4476	0.058*	
C13	−0.0930 (4)	0.4540 (3)	0.37347 (17)	0.0416 (8)	
C14	−0.1455 (4)	0.4348 (2)	0.31043 (18)	0.0457 (8)	
H14	−0.2145	0.4742	0.2882	0.055*	
C15	−0.0986 (4)	0.3585 (2)	0.27939 (16)	0.0410 (7)	
H15	−0.1342	0.3476	0.2360	0.049*	
C16	−0.1475 (5)	0.5364 (3)	0.4073 (2)	0.0593 (11)	
H16A	−0.2063	0.5125	0.4359	0.089*	
H16B	−0.2001	0.5785	0.3760	0.089*	
H16C	−0.0713	0.5713	0.4317	0.089*	
C17	0.2135 (3)	0.2461 (2)	0.24238 (14)	0.0326 (6)	
C18	0.2127 (4)	0.3395 (3)	0.22013 (16)	0.0411 (7)	
H18	0.1349	0.3781	0.2203	0.049*	
C19	0.3248 (4)	0.3757 (3)	0.19790 (17)	0.0475 (9)	
H19	0.3227	0.4387	0.1827	0.057*	
C20	0.4419 (4)	0.3201 (3)	0.19754 (17)	0.0509 (9)	
C21	0.4425 (4)	0.2289 (3)	0.21997 (18)	0.0529 (10)	
H21	0.5205	0.1907	0.2198	0.063*	
C22	0.3299 (3)	0.1914 (3)	0.24308 (16)	0.0428 (8)	
H22	0.3332	0.1289	0.2591	0.051*	
C23	0.5626 (4)	0.3616 (4)	0.1716 (2)	0.0784 (15)	
H23A	0.6203	0.3104	0.1608	0.118*	
H23B	0.6156	0.4021	0.2038	0.118*	
H23C	0.5292	0.3989	0.1337	0.118*	
C24	−0.0624 (3)	0.1663 (2)	0.20533 (14)	0.0314 (6)	
C25	−0.0789 (4)	0.2143 (3)	0.14741 (15)	0.0403 (7)	
H25	−0.0181	0.2635	0.1413	0.048*	
C26	−0.1852 (4)	0.1897 (3)	0.09838 (17)	0.0477 (9)	
H26	−0.1966	0.2236	0.0597	0.057*	
C27	−0.2745 (4)	0.1164 (3)	0.10531 (17)	0.0447 (8)	
C28	−0.2578 (4)	0.0687 (3)	0.1635 (2)	0.0516 (10)	
H28	−0.3182	0.0190	0.1693	0.062*	
C29	−0.1533 (4)	0.0933 (2)	0.21300 (17)	0.0416 (8)	
H29	−0.1438	0.0605	0.2521	0.050*	
C30	−0.3886 (6)	0.0899 (3)	0.0519 (2)	0.0748 (15)	
H30A	−0.4521	0.1428	0.0430	0.112*	
H30B	−0.4370	0.0348	0.0644	0.112*	
H30C	−0.3506	0.0749	0.0139	0.112*	

### Atomic displacement parameters (Å^2^)

**Table:**

	*U*^11^	*U*^22^	*U*^33^	*U*^12^	*U*^13^	*U*^23^
Au	0.03883 (7)	0.03027 (7)	0.02839 (7)	0.00283 (5)	0.00402 (5)	0.00126 (4)
S1	0.0541 (5)	0.0278 (4)	0.0343 (4)	0.0064 (4)	0.0052 (3)	0.0014 (3)
P1	0.0335 (4)	0.0303 (4)	0.0271 (4)	0.0013 (3)	0.0047 (3)	0.0002 (3)
O1	0.0516 (14)	0.0341 (12)	0.0317 (11)	0.0153 (11)	0.0043 (10)	0.0018 (9)
O2	0.0422 (15)	0.0540 (17)	0.101 (2)	0.0135 (13)	0.0091 (15)	0.0129 (16)
O3	0.0616 (19)	0.0425 (15)	0.111 (3)	0.0113 (14)	0.0197 (18)	0.0289 (17)
N1	0.0577 (18)	0.0352 (14)	0.0324 (14)	0.0134 (13)	0.0067 (12)	0.0042 (11)
N2	0.0456 (17)	0.0361 (15)	0.0510 (18)	0.0042 (13)	0.0011 (13)	0.0038 (13)
C1	0.0383 (16)	0.0299 (15)	0.0361 (16)	0.0056 (13)	0.0104 (13)	0.0024 (12)
C2	0.0501 (19)	0.0321 (15)	0.0269 (15)	0.0086 (14)	0.0028 (13)	0.0012 (12)
C3	0.0336 (17)	0.045 (2)	0.061 (2)	0.0051 (15)	0.0101 (15)	0.0110 (17)
C4	0.0371 (18)	0.0401 (18)	0.065 (2)	0.0030 (15)	0.0099 (16)	0.0149 (17)
C5	0.0377 (16)	0.0314 (15)	0.0365 (16)	0.0047 (13)	0.0032 (13)	0.0030 (12)
C6	0.0332 (16)	0.0428 (18)	0.051 (2)	0.0022 (14)	0.0083 (14)	0.0067 (15)
C7	0.051 (2)	0.0354 (18)	0.051 (2)	0.0025 (15)	0.0145 (17)	0.0067 (14)
C8	0.071 (3)	0.045 (2)	0.0354 (18)	0.0288 (19)	0.0093 (17)	−0.0038 (15)
C9	0.082 (3)	0.044 (2)	0.048 (2)	0.013 (2)	−0.005 (2)	−0.0100 (17)
C10	0.0330 (15)	0.0312 (14)	0.0298 (15)	−0.0013 (12)	0.0067 (12)	−0.0023 (11)
C11	0.0438 (19)	0.0458 (19)	0.0340 (17)	0.0032 (16)	0.0011 (14)	−0.0041 (14)
C12	0.058 (2)	0.052 (2)	0.0322 (18)	−0.0004 (18)	0.0020 (16)	−0.0125 (16)
C13	0.051 (2)	0.0346 (16)	0.0429 (19)	−0.0062 (15)	0.0174 (15)	−0.0069 (14)
C14	0.057 (2)	0.0379 (18)	0.042 (2)	0.0098 (16)	0.0104 (17)	0.0057 (14)
C15	0.0490 (19)	0.0401 (17)	0.0323 (16)	0.0078 (15)	0.0026 (14)	0.0017 (14)
C16	0.083 (3)	0.043 (2)	0.056 (2)	0.007 (2)	0.024 (2)	−0.0088 (18)
C17	0.0310 (14)	0.0390 (17)	0.0274 (14)	−0.0018 (13)	0.0036 (11)	−0.0034 (12)
C18	0.0414 (18)	0.0430 (18)	0.0401 (18)	−0.0018 (15)	0.0102 (14)	−0.0003 (14)
C19	0.049 (2)	0.051 (2)	0.043 (2)	−0.0126 (18)	0.0081 (15)	0.0011 (16)
C20	0.0401 (19)	0.077 (3)	0.0352 (18)	−0.0160 (19)	0.0047 (14)	−0.0061 (18)
C21	0.0342 (18)	0.075 (3)	0.049 (2)	0.0056 (18)	0.0062 (15)	−0.003 (2)
C22	0.0390 (18)	0.0475 (19)	0.0409 (18)	0.0039 (15)	0.0042 (14)	−0.0020 (15)
C23	0.042 (2)	0.117 (5)	0.079 (3)	−0.022 (3)	0.017 (2)	0.005 (3)
C24	0.0334 (15)	0.0290 (14)	0.0308 (15)	−0.0006 (12)	0.0030 (12)	−0.0021 (11)
C25	0.0420 (17)	0.048 (2)	0.0300 (16)	−0.0092 (15)	0.0043 (13)	0.0012 (14)
C26	0.053 (2)	0.054 (2)	0.0330 (18)	−0.0001 (18)	−0.0018 (15)	0.0019 (15)
C27	0.049 (2)	0.0404 (18)	0.0399 (19)	−0.0039 (16)	−0.0047 (15)	−0.0041 (15)
C28	0.053 (2)	0.042 (2)	0.054 (2)	−0.0173 (17)	−0.0057 (18)	−0.0004 (16)
C29	0.050 (2)	0.0349 (17)	0.0367 (18)	−0.0044 (15)	−0.0002 (15)	0.0051 (13)
C30	0.076 (3)	0.077 (3)	0.059 (3)	−0.023 (3)	−0.023 (2)	0.003 (2)

### Geometric parameters (Å, º)

**Table:**

Au—P1	2.2611 (8)	C13—C16	1.510 (5)
Au—S1	2.3105 (8)	C14—C15	1.380 (5)
S1—C1	1.756 (3)	C14—H14	0.9400
P1—C10	1.816 (3)	C15—H15	0.9400
P1—C24	1.809 (3)	C16—H16A	0.9700
P1—C17	1.815 (3)	C16—H16B	0.9700
O1—C1	1.354 (4)	C16—H16C	0.9700
O1—C8	1.457 (4)	C17—C22	1.382 (5)
O2—N2	1.215 (4)	C17—C18	1.395 (5)
O3—N2	1.211 (4)	C18—C19	1.374 (5)
N1—C1	1.259 (4)	C18—H18	0.9400
N1—C2	1.407 (4)	C19—C20	1.397 (6)
N2—C5	1.460 (4)	C19—H19	0.9400
C2—C7	1.381 (5)	C20—C21	1.366 (6)
C2—C3	1.389 (5)	C20—C23	1.514 (5)
C3—C4	1.379 (5)	C21—C22	1.395 (5)
C3—H3	0.9400	C21—H21	0.9400
C4—C5	1.375 (5)	C22—H22	0.9400
C4—H4	0.9400	C23—H23A	0.9700
C5—C6	1.377 (5)	C23—H23B	0.9700
C6—C7	1.379 (5)	C23—H23C	0.9700
C6—H6	0.9400	C24—C29	1.391 (5)
C7—H7	0.9400	C24—C25	1.387 (4)
C8—C9	1.510 (6)	C25—C26	1.389 (5)
C8—H8A	0.9800	C25—H25	0.9400
C8—H8B	0.9800	C26—C27	1.379 (5)
C9—H9A	0.9700	C26—H26	0.9400
C9—H9B	0.9700	C27—C28	1.391 (5)
C9—H9C	0.9700	C27—C30	1.502 (5)
C10—C11	1.379 (4)	C28—C29	1.384 (5)
C10—C15	1.378 (4)	C28—H28	0.9400
C11—C12	1.382 (5)	C29—H29	0.9400
C11—H11	0.9400	C30—H30A	0.9700
C12—C13	1.378 (6)	C30—H30B	0.9700
C12—H12	0.9400	C30—H30C	0.9700
C13—C14	1.377 (5)		
			
P1—Au—S1	175.80 (3)	C15—C14—H14	119.5
C1—S1—Au	100.18 (11)	C13—C14—H14	119.5
C10—P1—C24	105.90 (14)	C14—C15—C10	120.8 (3)
C10—P1—C17	103.93 (14)	C14—C15—H15	119.6
C24—P1—C17	107.71 (14)	C10—C15—H15	119.6
C10—P1—Au	110.22 (10)	C13—C16—H16A	109.5
C24—P1—Au	114.24 (10)	C13—C16—H16B	109.5
C17—P1—Au	114.04 (10)	H16A—C16—H16B	109.5
C1—O1—C8	116.2 (2)	C13—C16—H16C	109.5
C1—N1—C2	122.2 (3)	H16A—C16—H16C	109.5
O3—N2—O2	122.5 (3)	H16B—C16—H16C	109.5
O3—N2—C5	118.8 (3)	C22—C17—C18	118.9 (3)
O2—N2—C5	118.6 (3)	C22—C17—P1	119.7 (3)
N1—C1—O1	120.4 (3)	C18—C17—P1	121.3 (2)
N1—C1—S1	126.5 (3)	C17—C18—C19	120.4 (3)
O1—C1—S1	113.1 (2)	C17—C18—H18	119.8
C7—C2—N1	120.0 (3)	C19—C18—H18	119.8
C7—C2—C3	119.7 (3)	C18—C19—C20	120.9 (4)
N1—C2—C3	120.0 (3)	C18—C19—H19	119.6
C4—C3—C2	120.1 (3)	C20—C19—H19	119.6
C4—C3—H3	120.0	C21—C20—C19	118.4 (3)
C2—C3—H3	120.0	C21—C20—C23	122.1 (4)
C3—C4—C5	119.2 (3)	C19—C20—C23	119.5 (4)
C3—C4—H4	120.4	C20—C21—C22	121.4 (4)
C5—C4—H4	120.4	C20—C21—H21	119.3
C6—C5—C4	121.5 (3)	C22—C21—H21	119.3
C6—C5—N2	119.1 (3)	C17—C22—C21	119.9 (4)
C4—C5—N2	119.4 (3)	C17—C22—H22	120.0
C5—C6—C7	119.0 (3)	C21—C22—H22	120.0
C5—C6—H6	120.5	C20—C23—H23A	109.5
C7—C6—H6	120.5	C20—C23—H23B	109.5
C6—C7—C2	120.4 (3)	H23A—C23—H23B	109.5
C6—C7—H7	119.8	C20—C23—H23C	109.5
C2—C7—H7	119.8	H23A—C23—H23C	109.5
O1—C8—C9	110.2 (3)	H23B—C23—H23C	109.5
O1—C8—H8A	109.6	C29—C24—C25	118.8 (3)
C9—C8—H8A	109.6	C29—C24—P1	117.9 (2)
O1—C8—H8B	109.6	C25—C24—P1	123.2 (2)
C9—C8—H8B	109.6	C26—C25—C24	120.1 (3)
H8A—C8—H8B	108.1	C26—C25—H25	119.9
C8—C9—H9A	109.5	C24—C25—H25	119.9
C8—C9—H9B	109.5	C27—C26—C25	121.4 (3)
H9A—C9—H9B	109.5	C27—C26—H26	119.3
C8—C9—H9C	109.5	C25—C26—H26	119.3
H9A—C9—H9C	109.5	C26—C27—C28	118.3 (3)
H9B—C9—H9C	109.5	C26—C27—C30	121.0 (4)
C11—C10—C15	118.3 (3)	C28—C27—C30	120.6 (4)
C11—C10—P1	118.5 (2)	C29—C28—C27	120.9 (3)
C15—C10—P1	123.1 (2)	C29—C28—H28	119.6
C12—C11—C10	120.5 (3)	C27—C28—H28	119.6
C12—C11—H11	119.7	C28—C29—C24	120.5 (3)
C10—C11—H11	119.7	C28—C29—H29	119.8
C13—C12—C11	121.2 (3)	C24—C29—H29	119.8
C13—C12—H12	119.4	C27—C30—H30A	109.5
C11—C12—H12	119.4	C27—C30—H30B	109.5
C12—C13—C14	118.0 (3)	H30A—C30—H30B	109.5
C12—C13—C16	121.4 (3)	C27—C30—H30C	109.5
C14—C13—C16	120.5 (4)	H30A—C30—H30C	109.5
C15—C14—C13	121.0 (3)	H30B—C30—H30C	109.5
			
C2—N1—C1—O1	−179.4 (3)	C13—C14—C15—C10	1.7 (6)
C2—N1—C1—S1	0.4 (5)	C11—C10—C15—C14	−2.0 (5)
C8—O1—C1—N1	4.3 (5)	P1—C10—C15—C14	−179.3 (3)
C8—O1—C1—S1	−175.5 (3)	C10—P1—C17—C22	143.8 (3)
Au—S1—C1—N1	−165.6 (3)	C24—P1—C17—C22	−104.1 (3)
Au—S1—C1—O1	14.2 (2)	Au—P1—C17—C22	23.8 (3)
C1—N1—C2—C7	92.7 (4)	C10—P1—C17—C18	−33.7 (3)
C1—N1—C2—C3	−93.8 (4)	C24—P1—C17—C18	78.3 (3)
C7—C2—C3—C4	−2.1 (6)	Au—P1—C17—C18	−153.8 (2)
N1—C2—C3—C4	−175.6 (3)	C22—C17—C18—C19	1.5 (5)
C2—C3—C4—C5	0.4 (6)	P1—C17—C18—C19	179.1 (3)
C3—C4—C5—C6	1.3 (6)	C17—C18—C19—C20	−0.5 (5)
C3—C4—C5—N2	−178.6 (3)	C18—C19—C20—C21	−0.2 (6)
O3—N2—C5—C6	175.3 (4)	C18—C19—C20—C23	178.9 (4)
O2—N2—C5—C6	−4.3 (5)	C19—C20—C21—C22	−0.2 (6)
O3—N2—C5—C4	−4.8 (5)	C23—C20—C21—C22	−179.3 (4)
O2—N2—C5—C4	175.6 (4)	C18—C17—C22—C21	−1.9 (5)
C4—C5—C6—C7	−1.3 (5)	P1—C17—C22—C21	−179.5 (3)
N2—C5—C6—C7	178.6 (3)	C20—C21—C22—C17	1.3 (6)
C5—C6—C7—C2	−0.5 (6)	C10—P1—C24—C29	−96.7 (3)
N1—C2—C7—C6	175.7 (3)	C17—P1—C24—C29	152.6 (3)
C3—C2—C7—C6	2.2 (6)	Au—P1—C24—C29	24.8 (3)
C1—O1—C8—C9	85.1 (4)	C10—P1—C24—C25	80.7 (3)
C24—P1—C10—C11	159.0 (3)	C17—P1—C24—C25	−30.0 (3)
C17—P1—C10—C11	−87.6 (3)	Au—P1—C24—C25	−157.8 (3)
Au—P1—C10—C11	35.0 (3)	C29—C24—C25—C26	0.4 (5)
C24—P1—C10—C15	−23.7 (3)	P1—C24—C25—C26	−177.0 (3)
C17—P1—C10—C15	89.6 (3)	C24—C25—C26—C27	−1.4 (6)
Au—P1—C10—C15	−147.8 (3)	C25—C26—C27—C28	1.5 (6)
C15—C10—C11—C12	0.7 (5)	C25—C26—C27—C30	−179.6 (4)
P1—C10—C11—C12	178.1 (3)	C26—C27—C28—C29	−0.7 (6)
C10—C11—C12—C13	0.9 (6)	C30—C27—C28—C29	−179.5 (4)
C11—C12—C13—C14	−1.2 (6)	C27—C28—C29—C24	−0.3 (6)
C11—C12—C13—C16	177.3 (4)	C25—C24—C29—C28	0.5 (5)
C12—C13—C14—C15	−0.1 (6)	P1—C24—C29—C28	177.9 (3)
C16—C13—C14—C15	−178.6 (4)		

### Hydrogen-bond geometry (Å, º)

Cg1 and Cg2 are the centroids of the C10–C15 and C17–C22 rings, respectively.

**Table:**

*D*—H···*A*	*D*—H	H···*A*	*D*···*A*	*D*—H···*A*
C4—H4···*Cg*1^i^	0.94	2.64	3.528 (4)	157
N2—O2···*Cg*2^ii^	1.22 (1)	3.55 (1)	83.1 (2)	4 (1)
N2—O3···*Cg*2^ii^	1.21 (1)	3.83 (1)	70.2 (2)	4 (1)

Symmetry codes: (i) −*x*, −*y*, −*z*+1; (ii) −*x*+1, −*y*, −*z*+1.
